# Home-Based Gait Training Using Rhythmic Auditory Cues in Alzheimer's Disease: Feasibility and Outcomes

**DOI:** 10.3389/fmed.2019.00335

**Published:** 2020-01-31

**Authors:** Joanne E. Wittwer, Margaret Winbolt, Meg E. Morris

**Affiliations:** ^1^Physiotherapy Discipline, La Trobe Centre for Sport and Exercise Medicine Research, Faculty of Health Sciences, School of Allied Health, La Trobe University, Melbourne, VIC, Australia; ^2^Australian Institute for Primary Care & Ageing, La Trobe University, Melbourne, VIC, Australia; ^3^North Eastern Rehabilitation Centre, Melbourne, VIC, Australia; ^4^Academic and Research Collaborative in Health (ARCH), SHE College, La Trobe University, Melbourne, VIC, Australia

**Keywords:** Alzheimer's disease, auditory stimulation, gait, physiotherapy, music therapy

## Abstract

**Background/Purpose:** Although gait disorders occur early in the course of Alzheimer's disease (AD) and increase the risk of falling, methods to improve walking in the home setting are poorly understood. This study aimed to determine the feasibility of a home-based gait training program using rhythmic auditory cues for individuals living with mild to moderately severe AD.

**Methods:** Participants had probable AD with no other major conditions affecting locomotion. The intervention consisted of eight progressively modified 45-min gait training sessions delivered during home visits over 4 weeks. Experienced physiotherapists provided the therapy that incorporated rhythmic music cues for a range of locomotor tasks and ambulatory activities. On the days when the physiotherapist did not attend, participants independently performed a seated music listening activity. Walking speed, cadence, stride length, double limb support duration, and gait variability (coefficient of variation) were measured using an 8-m GAITRite® computerized walkway immediately before and after the physiotherapy intervention. Participant satisfaction was also assessed using a purpose-designed questionnaire.

**Results:** Eleven (median age, 77.0 years; median ACE III score, 66/100; 3 females and 8 males) community-dwelling adults living with AD participated. Wilcoxon signed rank tests revealed statistically significant increases in gait speed following the home-based physiotherapy intervention (baseline = 117.5 cm/s, post-intervention = 129.9 cm/s, *z* = −2.40, *p* < 0.05). Stride length also improved (baseline = 121.8 cm, post-intervention = 135.6 cm, *z* = −2.67, *p* < 0.05). There was no significant change in gait variability. The program was found to be feasible and safe, with no attrition. Participant satisfaction with the home-based music-cued gait training was high, and there were no adverse events.

**Conclusion:** A progressively modified gait training program using rhythmic auditory cues delivered at home was feasible, safe, and enjoyable. Music-cued gait training can help to reduce the rate of decline in gait stride length and speed in some individuals living with AD.

**Trial Registration:** http://www.anzctr.org.au/Default.aspx, ACTRN12616000851460.

**Universal Trial Number:** U1111-1184-5735.

## Introduction

Alzheimer's disease (AD) is characterized by a gradual decline in walking speed and balance, as well as eventual cognitive impairment (1–3). The characteristic short-stepped, slow-walking pattern seen in people living with AD can be accompanied by increased variability of step timing and foot placement (3, 4), thought to be related to decreased executive function (5). Fall risk also increases as AD progresses (6), particularly when people are recently discharged home from hospital (7). Reduced functional ability (8, 9) and admission to residential care (10, 11) occur often. Poor walking performance is correlated with the severity of dementia in AD (12, 13).

Despite recent reports of declining incidence of dementia in some areas, the number of people with dementia worldwide is still likely to increase, driven mainly by improved life expectancy (14). Prevalence estimates for 2040 are now at 90.3 million (15), and managing dementia is a priority for many health services. In the absence of a cure, there is a need to develop a range of interventions, including home-based physical therapies, to reduce the physical burden of care. Limited improvements in locomotion and balance in AD have been reported with pharmacotherapy approaches (16). To improve walking and balance and prevent falls, growing attention has been directed toward community-based allied health interventions for AD, such as exercise and music cues (17, 18).

Music therapy in its various forms has been applied to improve function and movement in people living with dementia. It has been shown to reduce behavioral symptoms as well as anxiety and depression (19, 20). Music therapy has also been used to enhance movement in people with dementia. Participation in group music listening sessions was associated with a decreased risk of falls in patients admitted to an acute hospital ward (21). The effects of music on movement are also evident as a motivator for engaging in exercise and physical activities (22, 23).

A specific application of rhythmic music to enhance the speed and consistency of movement applies principles of auditory-motor entrainment (24). This entails synchronization in time with a predictable auditory stimulus (e.g., rhythmic music). These rhythmic sounds aim to exploit the close connections between auditory and motor neural systems (25, 26). Possible mechanisms by which rhythmic auditory cues may have their effect on movement include mediating neurophysiological changes (25) and reducing movement variability (27). The availability of enhanced sensory information in the form of auditory cues may assist the learner to focus attention on the most relevant information to improve their motor performance (28).

Rhythmic music has been shown to improve walking speed and consistency for people with static neurological conditions, such as stroke (29, 30) and cerebral palsy (31). Similar effects have been shown for progressive degenerative conditions including multiple sclerosis (32) and Parkinson's disease (PD) (33, 34). Rhythmic music cueing has also been reported to reduce gait variability in PD (35).

Asking participants with dementia to consciously match their footstep rhythm to an auditory beat has produced mixed results (36, 37). Slower and more variable gait patterns were the instantaneous effects of a single session of walking in time to rhythmic music on gait patterns of a group of 30 people with mild to moderately severe AD (37). A nine-session gait training program over 2 weeks of walking in time to simple beat cues or rhythmic music also failed to show discernable benefits in a group of 28 people with severe dementia who were living in a long-term care facility (36).

In contrast, Pomeroy et al. used music-cued exercises to produce a significant improvement in mobility scores (including a gait item) in 16 participants with severe dementia in a residential facility (38). This suggests that disease severity may not necessarily be a barrier to effective music-cued gait training. Music-cued exercises have also been associated with social engagement, group participation, and enjoyment in people with AD (39, 40). Most of these trials have been conducted in residential aged care or hospital settings. There have been few investigations of home-based physical therapies for people living with AD, even though they are potentially more accessible for participants and their caregivers.

This current study aimed to assess the feasibility and outcomes of home-based, music-cued gait exercises, to inform the design and planning of a larger randomized controlled trial. Specifically, we explored the benefits and limitations of a home visit-based, progressive, multisession physiotherapy gait training program using rhythmic auditory cueing, on footstep patterns in community-dwelling people living with AD.

## Methods

### Study Design

We used a pre–post study design to evaluate the feasibility and preliminary outcomes of a physiotherapy gait rehabilitation program requiring synchronization of movement to rhythmic music. Ethical approval for the study was granted by the university ethics committee (HE16-016) and written informed consent was gained from all participants or their legally authorized representatives in accordance with the Declaration of Helsinki.

### Participants

A convenience sample of 11 participants was recruited from the caseload of a geriatrician in Melbourne, Australia. Invitation to participate required a diagnosis of probable AD confirmed at routine review by the geriatrician according to consensus criteria (41). To be included, participants needed to be able to walk 100 m independently without an assistive device, have adequate hearing to conduct a conversation (including with hearing aids), and the capacity to comprehend and follow instructions. Exclusion criteria included cardiac or pulmonary conditions or any neurological or musculoskeletal comorbidity or pain that would affect gait or balance. Participants required safety clearance in writing from their local medical practitioner to participate.

### Intervention

Two sessions of physiotherapy in the home at convenient times for each participant and their caregivers were given every week for 4 consecutive weeks (total of eight sessions). Therapy was delivered by one of five experienced and registered physiotherapists using the largest area in the house (usually a living room or lounge room). Small furniture items were moved to create enough space. Hallways were also used for faster walking activities and for long or complex walking sequences. Sessions lasted for up to 1 h, including rest periods.

The typical content for the music-cued therapy sessions is outlined in Table 1. Activities were designed to be sufficiently flexible to accommodate different levels of gait ability. They were progressively modified each week to include different and more challenging cued gait tasks. Each training session commenced with seated warm-up activities followed by a range of different physical activities in standing or walking with varied cue types, music, and tempi (Table 1).

**Table 1:** Details of training session components, auditory cues, and activities included in the 4 week gait training program using rhythmic auditory cues.

**Rhythmic auditory cues**	**Activities—progressed as tolerated**
**Type:** Metronome software application for handheld digital music player (Pro Metronome® http://eumlab.com/pro-metronome/) Rhythmic Music—a variety of commercially available music files **Player:** All cues played via a handheld digital music player connected via WIFI® to a portable speaker **Music Selection Criteria:** An unchanging tempo with a clearly discernible rhythm in 2/4 or 4/4 meter Music pieces in a range of styles (classical, popular and marches) were varied through the sessions Participants were asked to nominate preferred selections to be used during each session. **Frequencies:** Commercial software (Tempo Magic Pro®) was used to manipulate music cue tempo without altering pitch Comfortable pace walking cadence 110% comfortable pace walking cadence 90% comfortable pace walking cadence	**Warm-up:** Seated toe/heel tapping/knee lifts/hand clapping/vocalizing (“1,2”) **Upright activities:** **Stepping** Forwards/side/back stepping Crossover steps Step-ups Sequences of stepping Add arm movements **Walking** Walk on the spot Straight line walking forwards/backwards/sideways Walking with turns forwards/backwards/sideways Walk stop and go Walk with turns/stop and go Add arm movements **Vocalize: (“la la” or song lyrics)** **Cue fading:** maintain tempo of rhythmic activity as cue is muted and then made audible again.

Auditory musical cues consisted of metronome beats and a variety of music files played via a handheld digital music player connected via WIFI® to a portable speaker (Ultimate Ears Boom 3 Portable Bluetooth Speaker®). Commercial software (Tempo Magic Pro®) was used to manipulate the cue tempo. Selection criteria for music pieces included an unchanging tempo with a clearly discernible rhythm in 2/4 or 4/4 m, with recorded tempo close to the usual walking cadence of each participant. Music pieces included a range of styles; classical [e.g., “Arrival of the Queen of Sheba” from Solomon (42)], popular [e.g., “I'm Gonna Be” (43)], and marches [e.g., “Radetzky March Op 228” (44)] were varied through the sessions. Participants used a structured format to nominate preferred selections to be used during each individual session. The music piece used during the testing sessions (“Pomp and Circumstance March No. 1 in D” by Elgar) was not used during the training sessions. The mean self-selected comfortable walking cadence from the baseline testing session for each individual was used as the comfortable pace tempo for cues during their first week of training. Subsequently, the cue tempo was increased (or decreased) according to individual participant ability.

During the first intervention session, each participant was provided with a small digital music player (SanDisk Clip Sport®) preloaded with rhythmic music chosen according to their personal preferences. They were asked to take the music player home and listen to the music using headphones while seated in a comfortable chair for at least 15 min every day. The player was set to play the tracks in random order. They were also instructed to focus on the rhythm of each track and then move their arms and/or tap their toes to the beat if they wished to. There is strong evidence to suggest that simply listening to rhythmic music activates motor areas of the brain (45). Each participant was given a practice diary to record these daily seated practice sessions. The diary consisted of a single page with a box grid of each date over the intervention period. Participants were asked to tick the box for the relevant day if they had done the practice and put a cross if they had not.

### Outcomes

Participants attended the La Trobe University Human Movement Laboratory on two occasions for assessment: immediately before (baseline) and after (retest) the 4-week home-based physiotherapy training sessions. They attended with their spouse or caregiver, and taxi transport was provided if required. On arrival, participants were given time to become accustomed to the laboratory environment before commencing testing. Demographic data and personal characteristics were recorded during the baseline session. Participants also completed the Addenbrooke's Cognitive Examination III, which assesses cognition (attention/orientation, memory, verbal fluency, language, and visuospatial abilities) and the Geriatric Depression Scale (46).

### Primary Outcomes

The primary outcome was gait, assessed by quantifying walking velocity, stride length, stride time, and the variability of these measures. Gait patterns were measured using an 8-m long GAITRite® computerized walkway (CIR Systems, Inc. 12 Cork Hill Rd, Franklin, NJ 07416). Measurements using this system have high reliability in people with AD (47). Participants walked on the mat under three conditions. First, they walked at a self-selected comfortable pace (“As if you were walking to the local shop”). The two other conditions (in random order) were walking in time to each of music (“Pomp and Circumstance March No. 1 in D,” Elgar), and metronome cues, with cue tempo set at each individual's comfortable pace cadence. One initial familiarization walk was followed by a further seven walks for all three conditions. Gait trials were commenced and finished 2 m beyond each end of the mat to remove acceleration and deceleration phases. Short rests between walks were taken as required. Trials were repeated if the participant spoke, veered off the side of the computerized mat, or became distracted.

### Feasibility

Feasibility of music-cued gait training for AD was assessed by examining participant safety (reports of soreness lasting longer than 48 h related to the intervention), adverse events (reports of falls or other adverse events during the intervention period), adherence (missed sessions and missed days of independent practice), retention (number of dropouts), and satisfaction with the program (questionnaire with a possible highest score of 55 indicating maximum satisfaction).

### Data Analysis

Gait data were processed according to a previously published method (37). Briefly, for each participant, individual walk speeds were compared under each condition. “Outlier” walks (i.e., with a speed of more than 10 cm/s above or below the median for all walks for each individual participant) were removed. All remaining walks were then combined for each condition as if they were from a continuous walk instead of several passes over the electronic mat. Strides were then removed from either the baseline or retest “continuous walk” to match the numbers of strides for baseline and retest tests. To illustrate, if a participant had 25 uncued strides available for analysis, 28 strides with music cues and 27 with metronome cues, 3 music-cued strides and 2 metronome-cued were removed from the end of each condition, leaving 25 strides under each condition.

Pace (speed and stride length), rhythm (step time), and variability (coefficient of variation) were tested under cued (music and metronome) and non-cued conditions. Effect sizes were calculated using *r* = *Z*/√*n* (48). Statistical analysis was conducted using IBM SPSS software^©^ (version 24), and statistical significance was set to *p* < 0.05.

## Results

### Feasibility

Eleven people with AD were recruited within a timeframe of 12 months. Participant characteristics are included in Table 2. All participants were within a normal weight range according to body mass index scores, and only three had reported falling in the previous year, two more than once. The participants all lived with spouses in their own homes, and they each completed all eight training sessions. There were no changes in medications over the course of the study. Participant safety evaluations recorded no reports of soreness lasting longer than 48 h related to the intervention. Moreover, there were no reports of adverse events during therapy and no falls during the intervention period. Retention in the trial was very high with no dropouts or attrition reported. Adherence was excellent, with no missed physiotherapy sessions and only a small number of missed days of independent practice (median = 4% of total sessions missed, range = 0–27%). The satisfaction with the program was also very good. The median total score for the satisfaction questionnaire was 53 (range, 42–55), where a possible highest score of 55 indicated maximum satisfaction with the music-cued therapy program.

**Table 2:** Participant characteristics (*N* = 11).

**Participant characteristic**	**Median value (interquartile range)**	**Range**
Age (years)	77 (71, 80)	57–90
Height (m)	1.67 (1.65, 1.75)	1.53–1.82
Weight (kg)	69.8 (58, 79)	44–85.2
Body mass index	22 (18, 22)	14–24
ACE III score (total score 100^*^)	66 (43, 81)	24–88
ACE III Fluency score (total score 14^ⱡ^)	6 (2, 8)	0–9
No. of medications	3 (2, 5)	1–7
GDS (total score 15^∧^)	2 (2, 3)	0–8
Time since diagnosis (weeks)	119 (5, 221)	1–266
No. of falls in previous 12 months	0 (0, 1)	0–3

* *NB 2 cut-offs 88 (100% sensitive, 96% specific) and 82 (93% sensitive, 100% specific)*.

ⱡ *Fluency domain relies heavily on executive function (49)*.

∧ *Total score > 5 points is suggestive of depression. Scores > 10 almost always indicate depression (46)*.

### Gait Outcomes

Wilcoxon signed rank tests revealed statistically significant improvements in gait speed following the physiotherapy intervention (baseline = 117.5 cm/s, post-intervention = 129.9 cm/s, *z* = −2.40, *p* < 0.05) and stride length (baseline = 121.8 cm, post-intervention = 135.6 cm, *z* = −2.67, *p* < 0.05) with large effect sizes. Ten of the 11 participants increased their walking speed, and nine increased stride length. The median number of strides per participant used for gait analysis was 52 (range, 35–92) with at least 30 strides included per participant (50). Table 3 shows mean comfortable pace gait velocity, stride time and stride length, and variability of each measure at baseline and retest assessments, together with change scores. At baseline, four participants walked more slowly than 1 m/s, a previously suggested cut-off point for increased risk of adverse health-related events in healthy older people (51). Two of these participants increased comfortable pace gait speed above this value following the training. The median increase in gait speed of 12.4 cm/s was close to a previously defined MDC value of 13 cm/s at the 95% confidence level (47).

**Table 3:** Spatiotemporal measures and variability before and after eight home-based gait training sessions cued with rhythmic auditory stimuli (*N* = 11).

**Gait measure**	**Baseline median value (IQ range)**	**Retest median value (IQ range)**	**Change in median value (IQ range)**	**Wilcoxon signed rank test**	**Effect size (*r* = z/√N)**
**Velocity (cm/s)**	**117.5 (94.7, 121.2)**	**129.9 (115.6, 137.5)**	**12.4 (3.2, 20.7)**	***p*** **= 0.016**	**0.51**
**Stride length (cm)**	**121.8 (116.8, 128.5)**	**135.6 (123.4, 142.3)**	**7.9 (4.3, 12.8)**	***p*** **= 0.008**	**0.57**
Stride time (s)	1.073 (1.055, 1.200)	1.073 (1.043, 1.114)	−0.035 (−0.063, −0.007)	*p* = 0.091	0.36
Music-cued velocity (cm/s)	115.8 (94.7, 127.0)	119.4 (108.4, 137.0)	3.2 (−6.2, 14.4)	*p* = 0.328	0.21
Metronome-cued velocity (cm/s)	114.0 (93.2, 134.4)	136.2 (107.6, 137.4)	3.9 (−1.2, 12.1)	*p* = 0.213	0.27
Velocity variability (%)	3.92 (3.02, 4.33)	3.48 (2.94, 4.16)	−0.39 (−0.68, 0.36)	*p* = 0.424	0.17
Stride length variability (%)	3.00 (2.30, 3.54)	3.41 (2.00, 3.68)	0.07 (−0.39, 0.36)	*p* = 0.859	0.04
Stride time variability (%)	2.42 (1.99, 2.71)	2.23 (1.74, 2.68)	−0.13 (−0.32, −0.09)	*p* = 0.374	0.19

*Values in bold indicate significant improvement in performance*.

There was no significant change in gait variability following the 4-weeks program. This applied to variability in stride time, stride length, and velocity. Variability values were low at baseline and remained so, in agreement with previous AD gait studies (37, 50, 52).

Table 3 also shows the change from baseline to retest in gait speed when synchronizing footsteps to auditory cues. A comparison between uncued and cued gait speed at baseline showed a small instantaneous deterioration with both music and metronome cues, in line with previous findings (37). At retest, while cued walking tended to be slower with music cues, the median velocity when cued with a metronome was greater than uncued walking speed.

Of the five participants who walked more slowly than cognitively healthy people of similar age at baseline, four increased their comfortable pace speed after the intervention. Of the six participants who walked faster than previously reported normative values, four increased their comfortable walking speed further.

## Discussion

There is a growing body of research literature reporting the benefits of rhythmic music cues for improving movement and well-being in people living with AD (53, 54). A Cochrane review (55) has also shown benefits for people with dementia in general. Interactive music interventions are associated with increased social engagement (56–58), interactions with significant others (57), and health-related quality of life in people with AD (58). Raglio et al. (59) also showed that music therapy interventions can reduce agitation in some people living with dementia.

The current trial has demonstrated that music-cued physiotherapy can also be successfully implemented for some people who are in the early stages of AD. Despite the debilitating effects of this progressive neurological condition, individuals with AD were able to increase their walking speed when they participated in a 4-week, home-based, music-cued therapy program. The home-based gait training program using rhythmic music cues was also associated with high levels of safety, compliance, adherence, and satisfaction. These results support previous findings of the benefits of home-based exercise interventions for people with dementia (60–62).

The effectiveness of any intervention depends not only on whether it is beneficial and improves an individual's function and well-being but also its feasibility. Training sessions need to be accessible to the individual and their family and easy to schedule into weekly activities as well as being enjoyable. All participants in the current trial reported that they enjoyed the music-cued gait exercises and indicated that they would continue using their personal digital music players for independent practice after the research trial concluded. They were particularly positive about receiving the therapy at home, which removed the requirement of organizing travel to a center-based program and provided a familiar environment.

Our study found improvements in walking speed and stride length; however, no statistically significant reduction in gait variability. A strong relationship has previously been reported between gait variability and falls risk in people with AD (63, 64); however, the clinical significance of a reduction in gait variability in chronic diseases remains unclear. At baseline, participants in our study walked with low temporal and spatial gait variability, similar to normative values (65). The confidence to join a gait training study may be related to having better motor skills. Further reduction in variability of gait measures following the intervention was therefore unlikely.

The neural mechanisms by which music-cued gait training can improve motor control in AD are not completely understood; however, recent neuroimaging studies including functional magnetic resonance imaging and electroencephalography (EEG) have shed some light on mechanisms and brain areas involved (66, 67). A study of the effects of augmented visual and auditory feedback on learning an upper limb motor task reported that the auditory feedback group took longer to equal the performance of the visual feedback group but retained the skill better when the feedback was removed (67). A gradual reduction in reliance on feedback in the auditory group was reflected in the increased prefrontal involvement that suggested a greater initial cognitive load. This may be due to auditory stimuli being less useful in guiding performance compared to visual (67). This decrement in performance with initial exposure to auditory stimuli has previously been reported (37) and was in evidence in the current study. During practice, auditory feedback may have been integrated with proprioceptive input, thus decreasing reliance on auditory cues (67). Removal of auditory feedback after training produced deactivation of the task-specific auditory brain areas (67). Auditory cues may make multimodal associative learning possible where otherwise only visual and motor learning would have occurred (68).

Direct current EEG records of cortical activation were used in another study to support the observed behavioral changes in musical proficiency on a digital piano (66). EEG results also showed the time course of cortical changes with emergence of auditory-sensorimotor brain plasticity within a few minutes of commencing training (66).

Despite their cognitive impairment, it is possible for people with AD to benefit from an approach to delivering physical interventions, which is tailored to their learning strengths (69). In addition to improved motor function demonstrated in our study, physical exercise with music has been shown to enhance cognitive function more than exercise alone (70). This was evidenced by subtle neuroanatomical changes seen in the brains of healthy older people (70). It is possible that similar changes could occur in some people living with dementia, which may arguably slow the degenerative process.

Our results support the previous findings of improved mobility following movement training using temporal features of music (38, 71). This varies from an early report by Clair and O'Konski (36) who reported that people with severe dementia did not improve gait measures with either of music or metronome-cued training. Possible explanations for this variation relate to differences in the way the music cues were used or the measurement procedures. Pomeroy (38) used a range of movement to music activities performed with minimal assistance and scored gait using a numerical scale, whereas Clair and O'Konski used walking practice supported by two caregivers and reported temporospatial gait measures. Another study which reported little benefit of gait training with rhythmic music cues had only one session of exposure to the cues (37). People with mild to moderate dementia severity may need more practice with the cues to gain long-lasting benefits (72). This suggestion is supported by the positive results following eight training sessions in the current study.

The strengths of this trial were that it was conducted in a convenient home setting, with participant involvement in music selection and therapy delivery. Assessments and interventions were carried out using methods designed to elicit optimal responses from people living with dementia in response to findings of previous studies of people with cognitive impairment, of the difficulty of eliciting consistent and optimal physical function (73). Strategies included use of familiar environments, implicit learning techniques including “learning by doing,” errorless practice, and creating a strong therapeutic relationship with the same therapist (69).

Limitations of this study included the small sample of convenience which limits generalizability of findings. Therapy was delivered for eight sessions; thus, the longer-term benefits of this enjoyable form of therapy were not evaluated. The relative benefits of metronome cueing compared to music cueing were not evaluated in detail, although a previous study found no consistent difference between effects the two different types of rhythmic cue in a group with AD (37). The relative contributions of the exercise elements and the cueing elements of the intervention were not partitioned out. In addition, we did not conduct an economic evaluation of home-based therapy or compare the costs and outcomes with outpatient or hospital gait training services for this sample of people with AD. This analysis would be an important element of a larger randomized trial.

## Conclusions

Supporting recent worldwide studies showing the benefits of music-cued exercises for people living with dementia (55, 74), a twice-weekly home-based physiotherapy program of music-cued gait training helped people with mild to moderate AD to walk faster. As well as being enjoyable, the therapy was feasible and safe to deliver at home and associated with high levels of compliance. Future studies need to determine long-term benefits of home gait training as well as the costs of delivery in different geographical regions.

## Data Availability Statement

The datasets generated for this study are available on request to the corresponding author.

## Author Contributions

JW was involved in the conception and design of the study, data acquisition, analysis and interpretation, and the drafting and critical revision of the article. MW was involved in the conception and design of the study, and critical revision of the article. MM was involved in the conception and design of the study, data analysis and interpretation, and the drafting and critical revision of the article.

### Conflict of Interest

MM was employed by the company North Eastern Rehabilitation Centre, Healthscope. The remaining authors declare that the research was conducted in the absence of any commercial or financial relationships that could be construed as a potential conflict of interest.
